# Amputation in crush syndrome: A case report

**DOI:** 10.1016/j.ijscr.2020.05.087

**Published:** 2020-06-12

**Authors:** María Camila Arango-Granados, Diego Fernando Cruz Mendoza, Alexander Ernesto Salcedo Cadavid, Alberto Federico García Marín

**Affiliations:** a Fundación Valle del Lili, Cra. 98 ## 18-49, Cali, Valle del Cauca, Colombia; b Universidad Icesi, Cl. 18 #122-135, Facultad de Medicina, Cali, Valle del Cauca, Colombia

**Keywords:** CS, Crush syndrome, CPK, Creatinine phosphokinase, Crush syndrome, Crush injuries, Amputation, Fasciotomy, Rhabdomyolysis

## Abstract

**Introduction:**

Crush syndrome (CS) is a condition with a high morbidity and mortality due to severe electrolyte disorders, circulatory dysfunction and multiple organ failure, secondary to severe rhabdomyolysis and reperfusion injuries. There is controversy about the role of fasciotomy in the treatment of compartment syndromes due to crush injuries and it is still unknown if early amputation has patient-centered benefits.

**Case presentation:**

This is a 29-year-old patient whose lower body was trapped for 50 h under a 40-meter landslide. Upon admission the left thigh was edematous and painful. Laboratories revealed a creatinine of 1.58 mg/dL, hyperkalemia, metabolic acidosis, hyperlactatemia and creatinine phosphokinase (CPK) of 88,700 U/L, suggesting CS. Despite fluid and bicarbonate infusion his renal function worsened, CPK rose and left thigh became more tense, so a fasciotomy was performed. He developed a distributive shock refractory to vasopressors, steroids and methylene blue so amputation was proposed. Two hours after amputation the vasopressor support was nearly withdrawn.

**Discussion:**

This case suggests a potential benefit of amputation in patients with CS and progressive deterioration despite aggressive resuscitation. It also invites to think if this is a decision that should be considered before the establishment or in the initial stages of the syndrome, even if the viability of the extremity is still questionable.

**Conclusion:**

The presence of risk factors for poor prognosis can favor amputation despite the apparent viability of the limb and the morbidity of losing an extremity.

## Highlights

• Crush syndrome (CS) produces severe electrolyte disorders, circulatory and multiple organ failure due to severe rhabdomyolysis and reperfusion injuries.
• To date, the main stem of management is aggressive fluid resuscitation.
• Fasciotomy for the treatment of compartment syndromes due to crush injuries is still controversial, and it is still unknown if early amputation has patient-centered benefits.
• This case suggests a potential benefit of amputation in patients with CS and progressive deterioration. It also invites to think if this is a decision to consider early in the course of the disease.
• The presence of risk factors for poor prognosis and the natural course of the disease can favor amputation despite the apparent viability of the limb and the morbidity of losing of an extremity.

## 1 Introduction

Crush syndrome (CS) is a condition with a high morbidity and mortality due to severe electrolyte disorders, circulatory dysfunction and multiple organ failure, secondary to severe rhabdomyolysis and reperfusion injuries [1]. Retrospective reviews of natural disasters describe an approximate mortality of 4.3–13.4% [2,3]. The lower extremities are the most frequently traumatized [2,4], and since these include the largest muscle groups, they can cause extensive rhabdomyolysis and a higher incidence of acute renal failure.

There is still little evidence-based literature to guide the management of traumatic rhabdomyolysis and reperfusion syndrome, and it is unknown if early amputation has patient-centered benefits that merit the morbidity of losing an extremity. We therefore sought to describe a case that addresses this question. This work is in line with the Updating consensus Surgical CAse REport (SCARE) guidelines [5]. Written informed consent was obtained from the patient for publication of this case report and accompanying images.

## 2 Case presentation

**Fig. 1:**
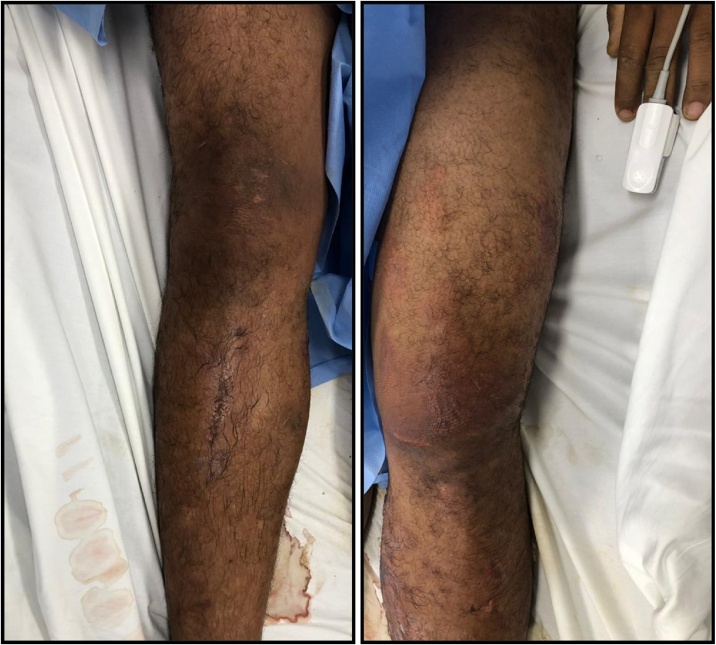
Lower extremities of a patient victim of a crush injury. The left thigh is edematous, with an increased perimeter with respect to the contralateral.

This is a 29-year-old patient who was trapped for 50 h under a 40-meter landslide that fell over his lower body. Upon admission he had a heart rate of 150 beat per minute, was normotensive and had multiple abrasions and blisters. The left thigh was edematous, painful, with an increased perimeter with respect to the contralateral (Fig. 1). The right heel had complex wound with exposed nerves and tendons but no bleeding. Laboratories revealed a hemoglobin of 21.2 g/dL, creatinine 1.58 mg/dL, potassium 6.11 mmol/L, serum pH 7.3, bicarbonate 16.6 mmol/L, lactic acid 4.68 mmol/L and a creatinine phosphokinase (CPK) of 88700 U/L.

**Fig. 2:**
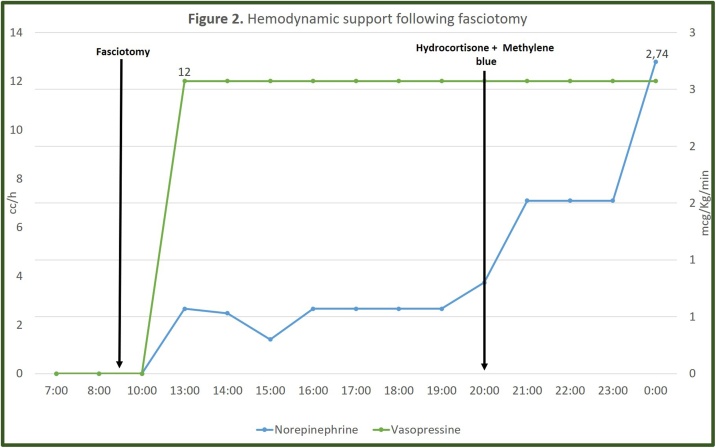
Hemodynamic support following fasciotomy.

Despite fluid therapy and bicarbonate infusion his renal function worsened and CPK rose (94894 U/L), and renal replacement therapy was needed. The left thigh became more tense and painful, so a fasciotomy was performed. In the immediate postoperative period, he developed a distributive shock. He continued with hyperkalemia (5.59 mmol/L), hyperlactatemia (3.98 mmol/L), metabolic acidosis, worsening of renal function (creatinine 2.64 mg/dL) and hypotension refractory to multiple vasopressors, steroids and methylene blue (Fig. 2). He was also receiving blood products due severe anemia, trauma induced coagulopathy (thromboelastogram with prolonged R and K times) and severe thrombocytopenia (platelets 38,000 × 10^3^ U/L).

**Fig. 3:**
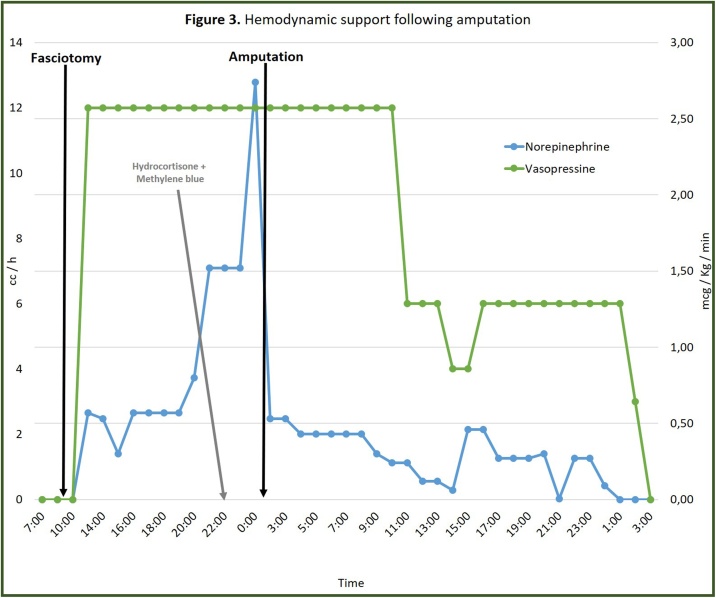
Hemodynamic support following amputation.

By that time (24 h after admission), the left lower extremity was distally cold and cyanotic, so the possibility of disarticulating the affected limb was discussed. Above-knee amputation was performed. Histologic descriptions of the extremity included extensive hemorrhage, acute inflammatory infiltrates and areas of ischemic necrosis. Two hours after amputation, vasopressin and norepinephrine were reduced by 50% and 90% of the maximum dose received, respectively, until the complete withdrawal of vasopressor support was achieved (Fig. 3).

The patient continued his improvement: he required 2 additional sessions of hemodialysis before complete resolution of the acute renal failure. He was transferred to general wards 13 days after admission to start rehabilitation and to continue the management of the complex wound on his right heel. He was finally discharged 47 days later with a rehabilitation plan.

## 3 Discussion

Crush syndrome was described during World War I and II by Seigo Ninami, a Japanese dermatologist, and Eric Bywaters, a British doctor. The first described the pathogenesis of this clinical condition; the second was the first to frame all these alterations and call them crush syndrome [6]. For this reason, this entity is also known as *Bywaters Syndrome*.

This phenomenon is common in natural and man-made disasters. However, there are other mechanisms, such as the prolonged crushing of the limbs due to an altered state of consciousness, prolonged surgery positions, certain drugs, and toxins. There is also pseudo-crush syndrome in patients in a situation of starvation (e.g., kidnap victims) who receive intermittent trauma with blunt objects, which produces a cumulative effect on muscle mass (that can be enhanced by dehydration) [1].

Trauma due to crushing of the extremities has three phases: the initial mechanical trauma, the period of ischemia and reperfusion. During compression and ischemia, the production of adenosine triphosphate is decreased and there is an intracellular influx of calcium, which activates proteases, phosphorylase and nucleases that initiate the muscle lysis. When the compression is longer than 1 h, crush syndrome is highly likely to develop. The muscle usually tolerates 2 h of “hot” ischemia; between 4 and 6 h, anatomical and functional changes begin and after 6 h there is cellular necrosis. However, in severe traumas the compression time necessary to produce the syndrome can be as short as 20 min, and tolerance to ischemia may be shorter [1].

Rhabdomyolysis produces massive release of potassium, phosphate, organic acids, myoglobin, CPK and thromboplastin. This explain all the alterations that can be observed during the syndrome, such as cardiotoxicity due to hyperkalemia, myoglobinuria and nephrotoxicity (cardinal signs), hypovolemia, hyperphosphatemia, hypocalcemia, metabolic acidosis and disseminated intravascular coagulation. In addition, the pathophysiology may progress to a distributive shock (or hemorrhagic in case of concomitant vascular trauma), acute respiratory distress syndrome and multiple organ failure due to a severe inflammatory response [1,7].

Finally, during reperfusion, phenomena such as the Odeh oxygen paradox (oxygen as a substrate for xanthine oxidase and other enzymes that produce free radicals) and calcium paradox (which enhances its intracellular influx) occur. Decompression can also cause pulmonary embolism due to abrupt release of thrombi, fat or bone marrow segments [1,7].

Many patients develop acute oligoanuric renal failure due to myoglobinuria. Descriptive and analytical studies on natural disasters have suggested that there is a positive correlation between CPK values and the duration of renal replacement therapy [8]. Logistic regression of a cohort of 2083 trauma patients in the Intensuve Care Unit, of which 85% had an abnormal CPK, suggests that age > 55 years, injury severity score (ISS) > 16 and CPK values > 5000 U/L are the main risk factors for the development of renal failure [9]. Another case-control study of patients with rhabdomyolysis and CPK > 1000 U/L found that admission creatinine is the main factor to predict the need for hemodialysis, with 1.7 mg/dL as the cut-off point with better predictive performance [10]. Other factors related to the development of renal failure are the number of injured limbs, dehydration on admission, metabolic acidosis, hypocalcemia and hyperuricemia [1].

The treatment of crush trauma begins with extrication since it is known that the risk of developing crush syndrome is proportional to the time of compression [11]. If the trapped limb prevents extrication, amputation can be considered at the scene. This strategy, as well as the use of tourniquets, may eventually prevent or delay reperfusion syndrome [1], although there is no objective evidence to support these recommendations.

To date, the main stem of management is aggressive fluid resuscitation. Infusion rates of 0.5–1.5 liters/h are proposed [1]. There is discussion about which are the most appropriate fluids for the hydration of these patients. Although expert consensus recommends avoiding solutions containing potassium (eg Ringer's Lactate) [11], its concentration in these solutions is low. Two randomized controlled clinical trials comparing saline solution with balanced crystalloids in acutely ill adults demonstrated a reduction in the composite outcome of death, renal replacement therapy or persistent renal injury with the use of balanced crystalloids compared to saline solution (SMART: 14.3% vs. 15.4%, P = 0.04; SALT-ED, 4.7% vs. 5.6%, P = 0.01) [12,13]. In addition, there is risk of hyperchloremic metabolic acidosis with the use of 0.9% saline in high quantities. For this reason, it is probably preferable to use balanced crystalloids for the resuscitation of these patients.

Traditionally, fluid resuscitation has been sought for diuresis targets of 2–3 mL/Kg/h until the clearance of myoglobinuria [14]. However, positive fluid balance has been linked to an increase in mortality [15,16]. There is growing evidence to suggest that fluid administration should be guided by perfusion parameters and volume response predictors [17]. So, it is likely that this should be the standard of care in patients with CS once the initial resuscitation is completed.

The use of sodium bicarbonate, acetazolamide or mannitol as strategies to increase myoglobin clearance is based on the pharmacokinetic and pharmacodynamic considerations of the drugs and on the pathogenesis of the disease. Nielsen et al. propose an algorithm for the management of traumatic rhabdomyolysis based on urine output and urinary and serum pH, which could help prevent or reduce renal injury [18]. However, there is still no solid evidence on the benefit of these treatment strategies and the possible adverse effects related to the administration of bicarbonate, acetazolamide or mannitol must be considered.

Much has been discussed about the role of fasciotomy in crush trauma. While it is suggested that early fasciotomy with decompression of all compartments is the treatment of choice in compartment syndrome [19], there is some controversy about the role of fasciotomy in the treatment of compartment syndromes that develop specifically after crush injuries. Most authors believe that due to the similarities between crush injury and compartment syndrome, they benefit from the same treatment, that is, early fasciotomy. Other authors, on the other hand, suggest that fasciotomies after crush injuries do not have good results and are directly related to a higher incidence of sepsis and dialysis requirement [3,20]. However, the origin of this controversy is based on the interpretation of descriptive reports of natural disasters that imply a very high risk of bias. To date there are no controlled clinical trials to stablish the impact of this intervention on crush trauma.

The first reports of amputations in crush injuries come from the descriptions of Bywaters [6]. In 1941 he described the case of a 17-year-old female who’s left leg had been buried for nine hours. The viability the limb was followed by measuring the circumference and through pulsations identified by an oscillometer. The author described how during the following 24 h after admission, even though the leg was apparently less edematous, the oscillometer readings fell. After that the leg appeared cold and blue below the knee, so amputation was proposed. Despite aggressive resuscitation, she died eight days after. The author then discussed if the timing in which amputation was performed could have impacted the results of the patient. In the same publication he told about a 34-year-old man who was trapped by beams across shoulders, arms, and thighs for twelve hours. Upon admission he was in shock. The next day his left arm was greatly swollen, with a blue, cold, and pulseless hand. A fasciotomy was performed and on the third day the arm remained warm. However, on the fourth day he turned febrile. During the following days fever persisted, his blood pressure fell, he became delirious and died on the seventh day.

To note, on both cases described by Bywaters [6] the decision to amputate was finally based on clinical objective evidence of limb death. Few data exist, if any, regarding which should be the indication to consider this intervention. The descriptions of clinical findings in the victims of Marmara earthquake (1999) reported 121 amputations in 98 patients, with a mortality of 30.5%. The presence or absence of amputations did not differ significantly in dialyzed vs non-dialyzed victims (p = 0.78) [3]. However, descriptions about management guidelines were not provided.

To date there are no controlled clinical trials evaluating the role of amputation in CS. What is most remarkable about the case presented is the hemodynamic response following amputation: two hours later, vasopressin and norepinephrine were reduced by 50% and 90% of the maximum dose received, respectively, until the complete withdrawal of vasopressor support was achieved. This, together with the patient’s outcome, suggest a potential benefit of this intervention in patients with CS and progressive deterioration despite advanced life support.

It is still unknown if early amputation has patient-centered benefits and it is likely that given the low frequency of this entity, many years and centers would be necessary to run a controlled clinical trial to solve this question. However, this case also invites to think whether this is a decision that should be considered early in the course of the disease, before the establishment or in the initial stages of the syndrome. Despite being a difficult decision for the medical group and for the patient, the presence of risk factors for poor prognosis and the natural course of the disease can favor amputation despite the apparent viability of the limb and the morbidity associated with the loss of an extremity.

## 4 Conclusion

Crush syndrome (CS) is a condition with a high morbidity and mortality due to severe electrolyte disorders, circulatory dysfunction and multiple organ failure, secondary to severe rhabdomyolysis and reperfusion injuries. To date, the main stem of management is aggressive fluid resuscitation, initially aiming adequate diuresis and subsequently guided by perfusion parameters and volume response predictors. The use of sodium bicarbonate, acetazolamide or mannitol as strategies to increase the myoglobin clearance is based on the pharmacokinetic and pharmacodynamic considerations of the drugs, and on the pathogenesis of the disease. There is controversy about the role of fasciotomy in the treatment of compartment syndromes due to crush injuries and it is still unknown if early amputation has patient-centered benefits. This case suggests a potential benefit of amputation in patients with CS and progressive deterioration despite advanced life support. The case also invites to think if this is a decision that should be considered early in the course of the disease, before the establishment or in the initial stages of the syndrome. Despite being a difficult decision for the medical group and for the patient, the presence of risk factors for poor prognosis and the natural course of the disease can favor amputation despite the apparent viability of the limb and the morbidity associated with the loss of an extremity.

## Declaration of Competing Interest

The authors declare no conflict of interests.

## Funding

No funding received

## Ethical approval

This case report was apporoved by the Ethics Committee of Fundación Valle del Lili Hospital (letter of approval 451-2019).

## Consent

Written informed consent was obtained from the patient for publication of this case report and accompanying images. A copy of the written consent is available for review by the Editor-in-Chief of this journal on request.

## Author contribution

MCA consolidated the information of the case, carried out the literature research and wrote the paper. DFC, AES and AFG participated in the treatment of the patient and contributed to the final version of the manuscript.

## Registration of research studies

1. Name of the registry: AMPUTATION IN CRUSH SYNDROME: AN INTERVENTION WORTH CONSIDERING
2. Unique identifying number or registration ID: researchregistry5491
3. Hyperlink to your specific registration (must be publicly accessible and will be checked): https://www.researchregistry.com/browse-the-registry#home/registrationdetails/5e8ca1928ba6fa001a402f4c/

## Guarantor

María Camila Arango-Granados.

## Provenance and peer review

Not commissioned, externally peer-reviewed.
